# Longitudinal development of the airway metagenome of preterm very low birth weight infants during the first two years of life

**DOI:** 10.1038/s43705-023-00285-x

**Published:** 2023-07-20

**Authors:** Ilona Rosenboom, Marie-Madlen Pust, Sabine Pirr, Alina Bakker, Maike Willers, Colin F. Davenport, Lutz Wiehlmann, Dorothee Viemann, Burkhard Tümmler

**Affiliations:** 1 Department for Pediatric Pneumology, Allergology and Neonatology, Hannover Medical School, Hannover, Germany; 2 Research Core Unit Genomics, Hannover Medical School, Hannover, Germany; 3 Translational Pediatrics, Department of Pediatrics, University Hospital Würzburg, Würzburg, Germany; 4 Cluster of Excellence RESIST (EXC 2155), Hannover Medical School, Hannover, Germany; 5 Center for Infection Research, University Würzburg, Würzburg, Germany

**Keywords:** Metagenomics, Metagenomics

## Abstract

Preterm birth is accompanied with many complications and requires severe therapeutic regimens at the neonatal intensive care unit. The influence of the above-mentioned factors on the premature-born infants’ respiratory metagenome or more generally its maturation is unknown. We therefore applied shotgun metagenome sequencing of oropharyngeal swabs to analyze the airway metagenome development of 24 preterm infants from one week postpartum to 15 months of age. Beta diversity analysis revealed a distinct clustering of airway microbial communities from hospitalized preterms and samples after hospital discharge. At nine and 15 months of age, the preterm infants lost their hospital-acquired individual metagenome signatures towards a common taxonomic structure. However, ecological network analysis and Random Forest classification of cross-sectional data revealed that by this age the preterm infants did not succeed in establishing the uniform and stable bacterial community structures that are characteristic for healthy full-term infants.

## Introduction

Preterm birth before the completion of 37 weeks of gestation is the leading cause of death for children until five years of age [1]. In 2014, it affected 10.6% of all pregnancies around the world and 8.7% in Europe [1]. With improvement of neonatal care, preterm infants of less than 28 weeks gestation now survive and the number of very low birth weight infants (VLBW, <1500 g) is increasing [2, 3]. Major complications of preterm birth are, among others, respiratory distress syndrome, necrotizing enterocolitis, sepsis, infections, retinopathy of prematurity, cerebral palsy and bronchopulmonary dysplasia (BPD) [4–7]. Several studies reported microbial dysbiosis in the airway microbiome of preterm neonates, who were to develop BPD, shortly after birth to one month postpartum [8–11]. With regard to the general consensus about the timing of bacterial colonization in the neonate [12, 13], initial colonization of the neonates’ airway is dependent on the mode of delivery [14]. Neonates born by vaginal delivery (VD) displayed bacterial communities similar to either the maternal vaginal and intestinal microbiota, whereas Caesarean section (CS) babies harbored skin and environmental microbiota [14]. Bosch et al. longitudinally analyzed the nasopharyngeal microbiome of healthy full-term neonates until six months of life. They reported differentiation into distinct microbial profiles at one week of age and delayed microbial turnover dynamics in CS neonates [15].

All aforementioned studies investigated the microbiome of healthy and BPD-affected infants with 16S rRNA gene sequencing. Whole genome shotgun sequencing overcomes the limitations of 16S rRNA gene sequencing as it profiles the taxonomic composition of complex bacterial, viral, fungal and archaeal communities down to species level [16, 17]. Whereas one study examined the airway metagenome of healthy infants and reported on the importance of both the high- (95% most abundant) and low-abundance (5% least abundant) species biosphere for shaping a healthy microbiome [18], to our knowledge, the longitudinal development of the airway metagenome of preterm infants has not been studied.

Therefore, this study investigated the development of the respiratory metagenome of 24 VLBW infants from one week postpartum to 15 months of age. For visualization, we also displayed cross-sectional data of healthy full-term controls who never received antibiotic therapy [18]. We considered oropharyngeal swabs as being representatives of the airway microbiome because the lower respiratory tract microbiome is reported to be more similar to the oral than the nasal communities due to microaspiration of microbes during sleep [19, 20].

We found that birth mode only significantly influenced the taxonomic composition of the respiratory metagenome one week after birth. At one month postpartum, we detected opportunistic pathogens and emerging respiratory commensals. Samples taken at the hospital were clearly distinguishable from those taken at nine and 15 months of age. While the overall community taxonomic profiles of preterms’ and full-terms’ metagenomes became comparable at late infancy, species co-occurrence networks demonstrated a different temporal evolution. Moreover, the taxonomic composition of the low-abundance taxa differed between preterm and full-term infants among Streptococci that constitute the major bacterial genus during infancy [18].

## Methods

### Study population

Twenty-four preterm neonates were recruited for our metagenome study (Table 1). They were born at Hannover Medical School (MHH) between 24 and 33 weeks of gestation. Oropharyngeal swabs for subsequent metagenome sequencing were collected one week (w1) and one month (m1) postpartum at the neonatal ICU (NICU) and at nine (m9) and 15 months (m15) of age during follow-up at the MHH outpatient clinic. Clinical metadata are provided in Supplementary Tables S1 and S2.

**Table 1:** Metadata of preterm study participants including information on longitudinal sampling.

Variables	Preterm infants
Number of study participants	24
Birth	
Median gestational age in weeks of pregnancy (range)	29 (24–33)
Median birth weight in grams (range)	958 (585–1910)
Median birth weight percentiles (in %)	40 (<3–70)
Mode of delivery	
Elective Caesarean section (CS)	6
Non-elective Caesarean section (CS)	11
Vaginal delivery (VD)	7
Female (in %) / Male (in %)	14 (58)/10 (42)
Median age at sampling in days postpartum (range)	
Sampling a (week 1, w1)	4 (2–14)
Sampling b (month 1, m1)	33 (29–40)
Sampling c (month 9, m9)	272 (252–308)
Sampling d (month 15, m15)	450 (422–503)
Number of samples	
Sampling a (w1)	23
Sampling b (m1)	24
Sampling c (m9)	23
Sampling d (m15)	17

To compare the development of the metagenome of the VLBW infants after hospital discharge, a control group of healthy full-term infants of similar age (median age 8 months, range 1–14 months) was recruited (*n* = 30). Cough swabs were collected during preventive medical examination or at kindergartens and local parent-child meetings in Hannover, Germany. Data regarding the healthy infants’ airway metagenomes were already published by our group [18]. The full-term infants’ clinical metadata including gender and age are summarized in Supplementary Table S3. VLBW infants’ and full-term infants' swabs were both identically processed and sequenced at MHH and were run through the same wet-lab and bioinformatics pipelines.

### Sample collection

Oropharyngeal swabs from our cohort of preterm infants born at Hannover Medical School in 2019 and 2020 were collected with sterile nylon-flocked swabs (Copan, Italy, #516 C). The preterm babies were longitudinally sampled at one week and one month postpartum and both at nine and fifteen months of age. Swab tips were placed in 2 ml tubes directly after trimming of the plastic handle with sterile scissors. Swabs were immediately stored at −20 °C and on the same day frozen at −80 °C until further processing.

The study was approved by the Ethics Committee of Hannover Medical School (no. 1510-2012, 9299_BO_K_2020, 9229_BO_S_2020). Informed consent was obtained from the parents or legal guardians prior to sample collection. Protocols were in accordance with the Helsinki Declaration of the World Medical Association and the General Data Protection Regulation of the European Union.

### Contamination control

To prevent DNA contamination, work areas and equipment were decontaminated with 2% (w/v) sodium hypochlorite solution [18]. During sample processing a sterile disposable coat, sterile gloves, face mask and hair cover were worn. Negative controls (blank swabs, working solutions) were processed and sequenced each time in parallel.

### DNA isolation, library preparation and sequencing

DNA was isolated as previously described [18]. Briefly, frozen swabs were soaked in 200 µl 0.1x TE buffer (Invitrogen, #12090015). A subsequent freezing-heating cycle of 5 min in a dry-ice-absolute-ethanol mixture and 3 min in a 65 °C heating block was repeated four times. The tubes were loaded onto an ultrasonicator (Covaris S220, program in [18]). Solution and swab were transferred to a manipulated 0.5 ml tube. A quick spin was performed to collect the DNA-containing solution. From that solution, 130 µl were pipetted in a Covaris microTUBE. The genomic DNA was sheared into 200–300 bp fragments [18] and subsequently purified with 1.2x AMPure XP beads (Beckman Coulter, #A63881). The Qubit dsDNA High Sensitivity Kit (Invitrogen, #Q32854) was used to measure DNA concentration. Fragment libraries of 20 ng input DNA were prepared without size selection using the NEBNext Ultra II DNA Library Prep Kit for Illumina (#E7645) and NEBNext Multiplex Oligos for Illumina (#E6440) with eight PCR cycles. The Illumina NextSeq 550 platform was used for sequencing of both patient samples and blank controls (NextSeq 500/550 High Output Kit v2.5, 75 cycles, #20024906).

### Bioinformatic processing: taxonomic classification and normalization

The whole metagenome sequencing pipeline Wochenende was used for quality filtering and aligning of the short reads against a reference database with default parameters (version 2.0.0) [21]. Next to one human genome as reference, the in-house curated database reviewed by clinical faculty contained one completely sequenced, quality-controlled genome per bacterial (*n* = 2228), viral (*n* = 38), fungal (*n* = 28) and archaeal (*n* = 212) species. Additional information and documentation for our pipeline can be retrieved from GitHub (https://github.com/MHH-RCUG/nf_wochenende/wiki). The reference database is available for download (https://drive.google.com/drive/folders/1q1btJCxtU15XXqfA-iCyNwgKgQq0SrG4). Normalization of microbial reads to bacterial cell per human cell was performed as previously described [22]. Included as a module, raspir filtered out species with non-uniform read distribution across a reference genome [23]. The R package metacoder was applied to generate heat trees [24]. We separately calculated the most (95%) and least (5%) abundant species, which we call high- and low-abundance taxa, respectively.

Whole genome shotgun sequencing and further processing, filtering and alignment of the reads with Wochenende [21] generated on the average 6.2 million high quality reads of 75 bp in length. The mean relative amount of microbial reads was 13%. Sequencing statistics as well as a relative abundance table are deposited in the GitHub repository (https://github.com/irosenboom/airway_metagenome_preterms).

### Statistical analysis

Statistical analyses were performed with R in RStudio version 2021.09.0. The Wilcoxon rank sum test (effect size *r*) and Kruskal–Wallis test (effect size epsilon-squared) were applied to compare two and more groups, respectively. Confidence intervals (ci) were obtained. Alpha and beta diversity of microbial communities were assessed by Shannon diversity (R function distance, R package *vegan*) [25] and NMDS of Bray–Curtis dissimilarity indices (R function metaMDS, *vegan*). A permutation test fitted clinical metadata and microbial species in the ordination (R function envfit, *vegan*). Group centroids were calculated with the betadisper function (*vegan*). A PERMANOVA was performed to determine how clinical metadata and metagenome variables explain the distance between the samples (R function Adonis, *vegan*).

Best practice guidelines were followed for the species co-occurrence network constructions [26]. Spearman’s rank correlation matrices were generated from bacterial cell per human cell-normalized and centered log-ratio (CLR) -transformed count data. All positive correlations were extracted (Spearman’s *p* value < 0.01, correlation coefficient ≥ +0.2). The open-source software *Gephi* *[*27] was utilized for an undirected network analysis with the continuous graph layout algorithm ForceAtlas [28]. The network parameters Degree centrality, Closeness centrality and Betweenness centrality were obtained. Degree centrality measures the numbers of connections of a node. Closeness centrality calculates the shortest distance of a node to all other nodes in the network [29], where a high value refers to a more central node. Betweenness centrality measures how often a node is bridged by the shortest pathway of two other nodes [29]. The Louvain algorithm was implemented to measure the division strength of networks into sub-communities in terms of modularity scores [30].

Random forest classification analyses based on bootstrapping aggregations were applied on cross-sectional data to identify the key determinants distinguishing same-aged ‘preterm infants’ from ‘healthy full-term infants’ both living in their family homes [31, 32]. Samples taken at the hospital were excluded from the analysis (w1, m1). Besides the airway metagenome variables (i.e. presence and abundance of species), the following variables were available: Antimicrobial therapy, gender, age (in days) and Shannon diversity. All non-random variables with mean decrease accuracy above zero were extracted. The classification performance was validated with the OOB estimate of error rate, class errors and the Boruta algorithm [33]. Random forest and Boruta wrapper application runs were repeated 100 times with different seeds for the classification and for the feature selection procedure with the objective to avoid a selection-based bias.

## Results

### Characteristics of the study participants

The 24 VLBW infants born at Hannover Medical School (MHH) in 2019 and 2020 between 24 and 33 weeks of gestation had a birth weight of 585–1910 g (Table 1). Six preterm neonates were born by elective Caesarean section (CS), eleven by non-elective CS and seven by vaginal delivery (VD). Twelve infants were diagnosed with BPD at 36 weeks of gestational age. All but one VLBW neonates received both breast milk and formula during their stay at the NICU (Supplementary Table S1). Oropharyngeal swabs were longitudinally collected at the NICU (w1, m1) and during follow-ups at our outpatient clinic after hospital discharge (m9, m15). The longitudinal sampling process is illustrated schematically as Supplementary Fig. S1. Clinical metadata are provided in Supplementary Tables S1 (delivery details, feeding) and S2 (medical treatment).

### Contamination control

DNA background contamination is a problem in metagenome sequencing of low-biomass habitats [34, 35]. Thus, we processed and sequenced both blank nylon swabs and water controls in parallel with patient samples in our study. After raspir filtering of species with a non-uniform distribution compared to their reference genome [23], no microbial reads were reported for 21 of 22 controls. The single bacterial read-positive control contained 93 reads of *Mycobacterium immunogenum* (Supplementary Fig. S2). *M. immunogenum* is associated with contaminated metalworking fluids [36]. We did not detect typical respiratory tract inhabitants.

### Microbiome composition and longitudinal development

**Fig. 1:**
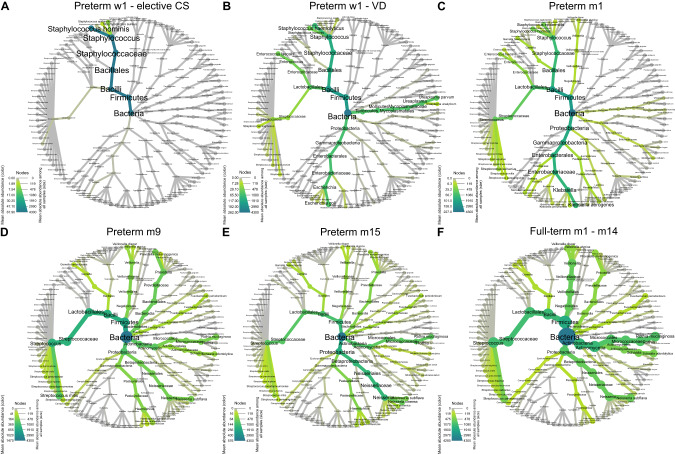
Representation of the upper airway bacterial metagenome development in the whole preterm cohort during the first 500 days of life and healthy full-terms in a heat tree format. The heat tree format represents mean absolute abundances per taxonomic rank from phylum to species. Node color indicates the bacterial load in the group, whereas node size allows a comparison to other groups in the same experiment. The gray background gives information on all species present in the whole cohort. **A** 1 week postpartum w1, elective CS, *n* = 6. **B** 1 week postpartum w1, vaginal delivery, *n* = 7. **C** 1 month postpartum m1, *n* = 24. **D** 9 months of age m9, *n* = 23. **E** 15 months of age m15, *n* = 17. **F** Healthy full-terms aged 37–398 days, *n* = 30.

Heat trees [25] were generated to visualize the taxonomic community composition of the airway metagenome during infancy (Fig. 1). The hierarchical heat tree format represents mean absolute abundances per taxonomic rank from phylum to species. Two of the six preterm neonates born by elective CS (*n* = 6) mainly harbored skin-associated Staphylococci, predominantly *Staphylococcus hominis* (Fig. 1A), whereas no microbial DNA was detected in samples from the other four preterm neonates. Besides Staphylococci, the preterm neonates born by VD (*n* = 7) were inhabited by typical members of the oral and gastrointestinal tracts, namely *Escherichia coli*, *Enterococcus faecalis* and various Streptococcus spp. (Fig. 1B). *Ureaplasma parvum* (Fig. 1B) was isolated from one patient whose mother had experienced preterm labor due to suspected intra-amniotic infection. One month postpartum typical commensals of the airway microbiome (*Veillonella atypica*, *Gemella haemolysans, Streptococcus salivarius*) and opportunistic pathogens like *Klebsiella aerogenes*, *Serratia marcescens* and *Raoultella ornithinolytica* were identified irrespective of the mode of delivery (Fig. 1C). At 9 months of age, after being home for at least half a year, the metagenome diversified into numerous commensals (Fig. 1D). *Streptococcus mitis*, *Prevotella melaninogenica*, *Rothia mucilaginosa*, *Neisseria subflava* and *Schaalia odontolytica* comprised the most abundant species. By 15 months of age, the overall taxonomic composition was maintained (Fig. 1E) rather similar to that of healthy full-term infants with a mean age of eight months (Fig. 1F).

### Alpha diversity

**Fig. 2:**
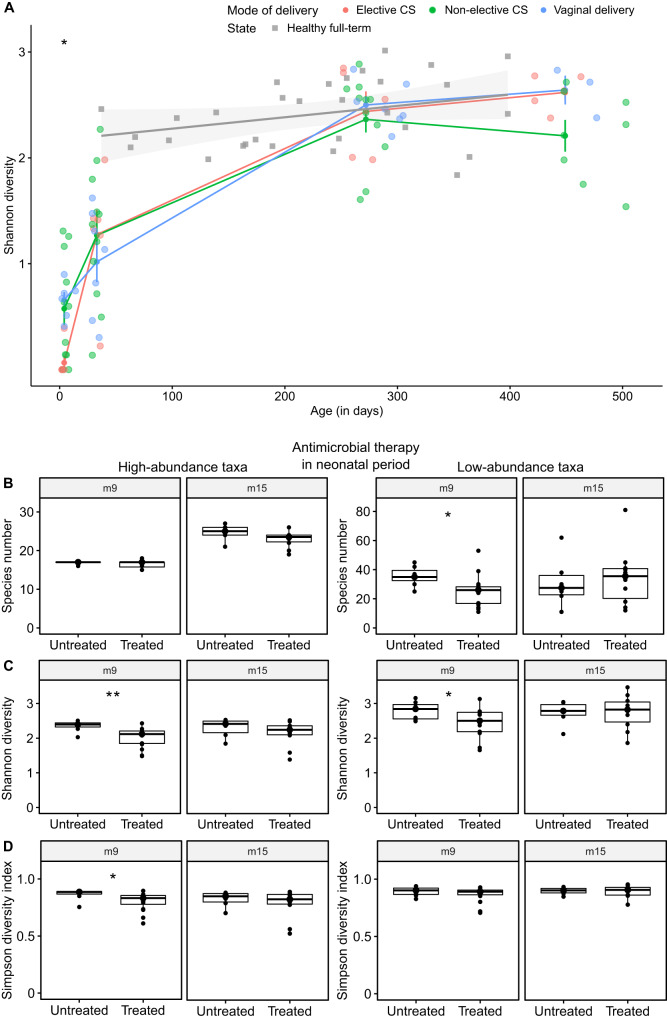
Alpha diversity. **A** Development of alpha diversity with age in the preterm cohort. Cross-sectional dataset of healthy full-term infants (gray) is displayed in the background. Elective CS, non-elective CS and VD are represented by red, green and blue dots, respectively. Shannon diversity was significantly different amongst preterm neonates by mode of delivery one week after birth (*p* value = 0.01, epsilon-squared effect size = 0.41, ci = 0.15–0.72), but not thereafter (m1, m9, m15: *p* value > 0.05). Error bars represent mean standard errors for every time point of sampling. **B** Antimicrobial treatment at the hospital significantly reduced the number of low-abundance taxa at 9 months postpartum (*p* value = 0.019, effect size *r* = 0.50, ci = 0.13–0.77). **C** Treatment with antibiotics during the stay at the neonatal ICU significantly reduced the Shannon diversity of the high-abundance (*p* value = 0.006, effect size *r* = 0.56, ci = 0.17–0.8) and low-abundance taxa (*p* value = 0.047, effect size *r* = 0.42, ci = 0.05–0.7) at 9 months postpartum, but not at 15 months postpartum anymore. **D** Antimicrobial therapy during the neonatal period significantly decreased the Simpson diversity of the high-abundance taxa (*p* value = 0.023, effect size *r* = 0.47, ci = 0.09–0.77). Sample sizes: m9 (untreated *n* = 7, treated *n* = 16), m15 (untreated *n* = 6, treated *n* = 10). Note: *p* values are depicted in the diagram with *p < 0.05 and **p < 0.01. Boxplots with medians are shown, the lower and upper boundary define the first and third quartiles (25th and 75th percentile, IQR). Whiskers extend to the largest and smallest non-outlier data points (1.5× IQR).

The development of Shannon diversity with age was investigated in our preterm cohort. Data are visualized in Fig. 2A along with a cross-sectional dataset of healthy full-term infants in the background. Irrespective of delivery mode, Shannon diversity increased in the preterm infants during the first nine months of life and was indistinguishable from that of healthy full-term infants thereafter. However, the oropharynx of preterm neonates born by elective CS initially harbored microbial communities of lower diversity (Kruskal–Wallis *p* value = 0.01, epsilon-squared effect size = 0.41) compared to non-elective CS and VD (Fig. 2A). While antimicrobial therapy during the postpartum stay at the hospital led to a significantly reduced Shannon diversity of the high-abundance (Wilcoxon *p* value = 0.006, effect size *r* = 0.56) and low-abundance (*p* value = 0.047, effect size *r* = 0.42) taxa by month 9, this effect was no longer apparent by month 15 (Fig. 2C). The significant influence of neonatal antimicrobial therapy indicated by a lower number of the low-abundance taxa (*p* value = 0.019, effect size *r* = 0.50) (Fig. 2A) and reduced Simpson diversity of the high-abundance taxa (*p* value = 0.023, effect size *r* = 0.47) (Fig. 2D) at m9 was restored six months later at the m15 time point.

### Beta diversity

**Fig. 3:**
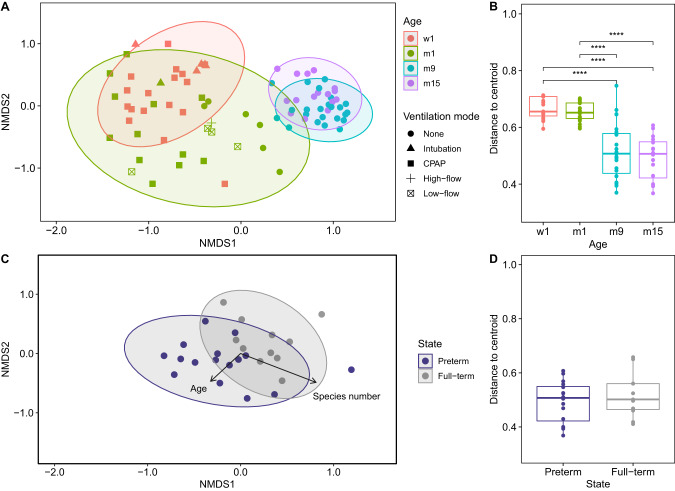
Beta diversity analysis of the preterm cohort and age-matched healthy full-term controls. **A** Investigation of longitudinal samples from the preterm cohort by applying two-dimensional non-metric multidimensional scaling to Bray–Curtis dissimilarity indices (stress = 0.18). Oropharyngeal swabs taken at the neonatal ICU are represented by red (w1) and green (m1) color. Samples collected at the outpatient clinic are depicted in blue (m9) and purple (m15). The shape of data points represents the ventilation mode. **B** Multivariate homogeneity of group variances differentiated by patients’ age revealed significant differences (*p* value < 0.0001, epsilon-squared effect size = 0.58, ci = 0.42–0.72). **C** NMDS based on Bray–Curtis dissimilarity index matrices (stress = 0.15) to compare the oropharyngeal metagenome of m15 preterm samples (blue, *n* = 17, 422–503 days of age) and age-matched healthy full-term controls (gray, *n* = 11, aged 269–398 days). **D** No significant differences were detected by Wilcoxon rank sum in multivariate homogeneity of preterm and term group variances. Note: Boxplots with medians are shown, the lower and upper boundary define the first and third quartiles (25th and 75th percentile, IQR). Whiskers extend to the largest and smallest non-outlier data points (1.5x IQR).

The beta diversity of the microbial communities of the healthy full-term and preterm infants was assessed by non-metric multidimensional scaling of Bray–Curtis dissimilarity indices (Fig. 3). The clustering of the longitudinal preterm samples was driven by infants’ age, number of species and Shannon diversity (Supplementary Table S4A). Ventilation during the neonatal period had a profound immediate influence on the community composition, as non-ventilated neonates at m1 were closest to the samples at m9 and m15 (Fig. 3A). While metagenome signatures segregated by patients’ residence at hospital and private home (Fig. 3A), they were more dissimilar in the shared hospital environment (w1 and m1 at NICU) than in their individual family environments (m9 and m15) (Fig. 3B). Samples obtained at the NICU exhibited significantly higher distances to their group centroid in multivariate space (beta dispersion) than samples obtained after hospital discharge, pointing towards an evolution of a common microbial community signature (Fig. 3B). When we compared the airway metagenome of our 15-month-old preterm infants with the only available metagenome dataset of age-matched healthy full-term infants, the two groups revealed distinct clustering mainly driven by the number of microbial species (Fig. 3C, Supplementary Table S4B). The preterm infants’ airways at this point of time were populated with communities distinct in beta diversity from their healthy peers evident in reduced numbers of microbial species. However, apparent in the homogeneity of beta dispersion, common metagenome signatures were retained (Fig. 3D). Consistent with the alpha diversity analysis, an influence of antimicrobial therapy was not detectable at 15 months of age (Supplementary Table S4B). A PERMANOVA confirmed the significant influence of the variables detected by the *envfit* permutation test, namely age, mode of ventilation and microbial diversity for the longitudinal preterm cohort and microbial richness for discrimination of same aged preterm and full-term infants’ airway metagenomes (Supplementary Table S5).

### Microbial community analysis by co-occurrence networks

**Fig. 4:**
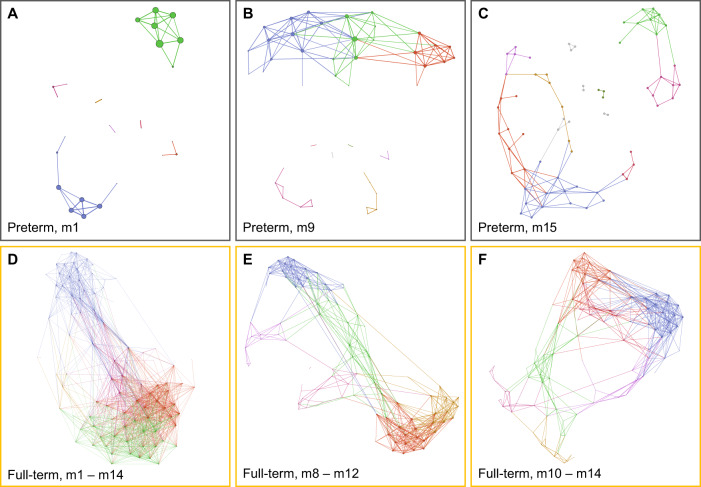
Ecological co-occurrence network analysis in preterms and healthy full-term controls. Spearman’s rank correlation matrices were obtained from bacterial cell per human cell-normalized and CLR-transformed count data. The software Gephi with the graph layout algorithm ForceAtlas [27] was applied to generate networks. Node size visualizes Degree centrality and color reflects the up to twelve modularity classes (1, blue; 2, green; 3, red; 4, pink; 5, orange; 6, light purple; 7, ruby; 8, dark green; 9, gray; 10, gray; 11, gray; 12, gray). **A** Co-occurrence network obtained from preterm neonates one month postpartum (*n* = 24). The two mostly contributing modules out of seven are depicted in blue (29.6%) and green (25.9%) based on decreasing network contribution. **B** Species co-occurrence network from randomly selected preterm infants at nine months of age (*n* = 13). The three mostly contributing modules out of nine are depicted in blue (23.5%), green (17.7%) and red (15.7%). **C** Species co-occurrence network from randomly selected preterm infants at 15 months of age (*n* = 11). The three mostly contributing modules out of twelve are depicted in blue (20.6%), green (13.2%) and red (13.2%). **D** Species co-occurrence network of healthy full-term controls between one and 14 months of age (n = 30). The three mostly contributed modules out of five contributed 34.7% (blue), 26.3% (green) and 23.2% (red). **E** Species co-occurrence network of healthy full-term controls between eight and twelve months of age (*n* = 13). The three mostly contributing modules out of seven are depicted in blue (21.1%), green (19.0%) and red (17.9%). **F** Species co-occurrence network of healthy full-term controls between ten and 14 months of age (*n* = 11). The three mostly contributing modules out of seven are depicted in blue (22.9%), green (16.7%) and red (14.6%).

We investigated the early development of bacterial co-occurrence networks in preterm infants in comparison to age matched healthy full-term infants (Fig. 4). Randomly selected preterm infants at m9 (*n* = 13) and m15 (*n* = 11) were contrasted with age- and sample size matched full-term infants. The number of contributing species (nodes) in the preterm network structure continuously increased until the last sampling time point at 15 months of age, but was almost twice as high in full-term infants. A decrease in network fragmentation was observed between one to 15 months of age as the number of contributing species and their connections (edges) continuously increased (Table 2). All preterm networks consisted of at least six sub-communities with species more closely connected to each other than to other species in the network (Fig. 4, Table 2). Between the first and fifteenth month of life, Degree centrality remained at the same low level in preterm networks. Although Closeness centrality and Betweenness centrality increased to nine months of age, they reverted to the m1 level thereafter at 15 months of age (Supplementary Fig. S3). Whereas the proportions of positive correlations increased from 87.1% at nine to 93.8% at 15 months of age, their proportions were still higher in the age-matched healthy peers (99.6% and 98.4%, Table 2). Full-term communities had a different structure; i.e., first, microbial species were more closely connected to each other as the number of edges was almost four times higher and second, Degree centrality was significantly increased (Fig. 4, Table 2, and Supplementary Fig. S3).

**Table 2:** Network parameters of the undirected graph analysis.

	w1	m1	m9	m15	Full-term (m8–m12)	Full-term (m10–m14)	Full-term (m1–m14)
Sample size	23	24	13	11	13	11	30
Number of nodes	0	27	51	68	95	96	95
Number of edges	0	35	118	124	517	481	1116
Average Degree	0	2.6	4.6	3.6	10.9	10.0	23.5
Modularity	0	0.66	0.54	0.68	0.58	0.53	0.33
Connected components	0	7	7	6	2	1	1
Number and contribution of modules	0	7 29.6% 25.9% 11.1% 11.1% 7.4% 7.4% 7.4%	9 23.5% 17.7% 15.7% 13.7% 11.8% 5.9% 3.9% 3.9% 3.9%	12 20.6% 13.2% 13.2% 11.8% 8.8% 7.4% 5.9% 4.4% 4.4% 4.4% 2.9% 2.9%	7 21.1% 19.0% 17.9% 13.7% 13.7% 12.6% 2.1%	7 22.9% 16.7% 14.6% 13.5% 11.5% 10.4% 10.4%	5 34.7% 26.3% 23.2% 9.5% 6.3%
Positive correlations		76.1%	87.1%	93.8%	99.6%	98.4%	99.7%

Force Atlas (Inertia = 0.1, Repulsion strength = 2000.0, Attraction strength = 1.0, Maximum displacement = 10, Auto stabilize function = TRUE, Gravity = 30).

### Random forest analysis

**Fig. 5:**
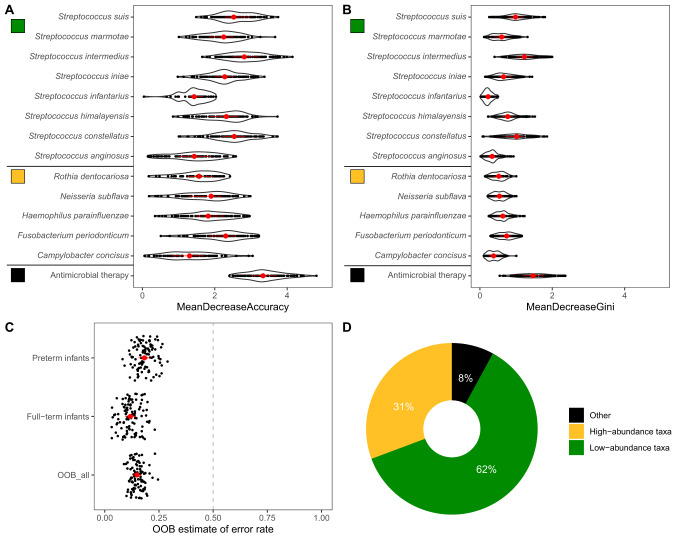
Random Forest classification analysis to predict clinical and taxonomic features associated with preterm and full-term delivery. Microbial taxonomy data, clinical metadata and diversity parameters were included in the model. **A** Representation of the classification outcome based on mean decrease accuracy. **B** Representation based on mean decrease Gini. **C** Overview of the out-of-bag (OOB) estimate of error rates for classifications, which were obtained from 100 times repeated Random Forest and Boruta wrapper application runs with different seeds. **D** Taxonomical and clinical (other) variables contributing as a predictor for classification as ‘preterm’ or ‘full-term’. The 95% most abundant species were classified as high-abundance taxa.

Feature selection and Random Forest bootstrapping aggregation were performed to identify the airway metagenome features and host- or treatment-associated variables distinguishing ‘full-term healthy infants’ from ‘preterm infants’ (Fig. 5). A low median out-of-bag (OOB) estimate of error rate of 0.15 (Fig. 5C) validated the performance of our Random Forest analysis. While antimicrobial therapy was, as expected, strongly associated with the delivery time point (full-term vs. preterm) (Fig. 5A, B), most of the factors contributing to the Random Forest decision were associated with the low-abundance taxa of the airway metagenome (62%, Fig. 5D). Various rare Streptococci such as *S. intermedius*, *S. infantarius* and *S. himalayensis* were less abundant in preterm infants (see primary data deposited at GitHub).

### Case report

**Fig. 6:**
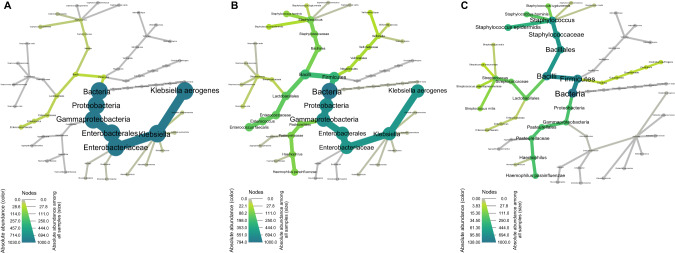
Case report of the airway metagenome of 29 days old triplets born by non-elective CS at 29 weeks of gestation. All neonates obtained support by low-flow ventilation. No antibiotic therapy was administered. We could detect three clearly separable metagenomes: Triplet1 shows a signature dominated by the nosocomial pathogen *Klebsiella aerogenes* (**A**), triplet2 has a more diverse respiratory microbiome, where *K. aerogenes* is also most abundant (**B**) and triplet3 is missing *K. aerogenes*, but additionally harbors *Haemophilus parainfluenzae* (**C**).

Finally, to stimulate the reader’s curiosity, we describe the unexpected variety of the metagenomes of preterm triplets who did not receive any antimicrobials during their stay at our NICU (Fig. 6). Despite their genetic relatedness, a matching therapeutic regimen and temporal stay at the NICU, their metagenomes 29 days postpartum were discordant. The metagenome of triplet 1 (female, 1.46 kg) was dominated by *K. aerogenes* (Fig. 6A), that of triplet 3 (female, 1.81 kg) was more diverse with *Staphylococcus epidermidis* and *Haemophilus parainfluenzae* being the two most abundant species (Fig. 6C). The microbial community of triplet 2 (male, 1.70 kg) presented as a mixture of his sisters’ metagenomes (Fig. 6B).

## Discussion

In this study, we applied shotgun metagenome sequencing to analyze the airway metagenome development of preterm neonates from one week postpartum to 15 months of age. Beta diversity analysis revealed distinct clustering of airway microbial communities from hospitalized VLBW neonates and samples after hospital discharge. At nine and 15 months of age, the preterm infants lost their hospital-acquired individual metagenome signatures and developed a common overall taxonomic structure. However, ecological network analysis and Random Forest classification of cross-sectional data revealed that by this age the preterm infants did not succeed in establishing the uniform bacterial community structures that are characteristic for healthy full-term infants.

Alpha diversity analysis revealed the significant impact of birth mode on the airway metagenome at one week postpartum. VLBW neonates born by elective CS were either sterile or harbored skin-associated Staphylococci, whereas vaginally delivered preterm neonates displayed vaginal and gastrointestinal microbiota indicating the seeding from the mother as has been reported previously in full-term infants [14, 15, 37]. In our study, this influence of the mode of delivery was no longer detectable one month postpartum: individual microbial signatures consisting of commensal and opportunistic pathogens emerged. Previous studies based on microbial culturing or 16S rRNA gene sequencing demonstrated that the hospital environment and therapeutic regimens shape individual microbial community structures of VLBW infants during their stay at the NICU [38–42]. In our preterm cohort, the mode of ventilation and antimicrobial therapy affected beta biodiversity, e.g., most microbial communities of non-ventilated infants were already quite close to those seen months later at home. Even though the effect of treatment regimen at the NICU on alpha and beta diversity was no longer apparent at 15 months of age, the bacterial co-occurrence networks in preterm infants still consisted of six fragments opposed to the single closely connected structure observed for healthy full-terms. Fragmented co-occurrence networks identified in preterm infants in this study have already been associated with dysbiosis in chronic lung diseases such as cystic fibrosis and bronchiectasis [43–46]. Random Forest classification taught us that the low-abundance taxa within the bacterial community structure are instrumental for the differentiation between ‘preterms’ and ‘full-terms’. Species of the genus Streptococcus are the dominant taxa in the emerging airway microbiome of healthy infants during the first year of life [18]. Interestingly, new Streptococcus species that recently received their names such as *S. himalayensis* in 2017 [47] and *S. marmotae* in 2016 [48] were prevalent in most swabs collected from 1-year old full-term infants but were only present in a minority of samples retrieved from age-matched VLBW infants. Hence, the presence of these novel rare Streptococcus species could be biomarkers for the differentiation of mature and immature microbiomes at the beginning of toddler age. At least during the first two years of life preterm and full-term infants still differ in their respiratory microbiome in the composition of the low-abundant bacterial taxa. Unexpectedly, we did not detect an influence of BPD on the preterm microbial metagenome at late infancy, which of course could manifest later in life. Furthermore, our intriguing case report on the neonatal triplets’ divergent metagenomes tells us that we should envisage a wide range of direct and indirect determinants most of which we are still unaware of that shape our airway microbiome in early life.

A limitation of this study is the comparably small cohort size of 24 preterm infants. Therefore, we report effect size to validate our statements. To avoid oral contamination, sampling by bronchoalveolar lavage or induced sputum is recommended for airway metagenome analyses [49]. However, in the context of our cohort of ventilated VLBW neonates only sampling by oropharyngeal swabs was ethically justifiable. Therefore, to maintain an identical sampling procedure within the preterm cohort, oropharyngeal swabs were taken also until early infant age.

We compared the respiratory metagenome of preterm infants sampled by oropharyngeal swabs with cough swabs retrieved from full-term infants. There is an agreement in the literature over a gradient of microbial load, richness and diversity from the upper to the lower healthy human airways [19, 50]. In our study, we observed similar microbial diversity, but reduced species richness in preterm oropharyngeal swabs compared to full-term cough swabs. Considering the aforementioned microbial gradient in the human airways, the differences we observed would have been even more striking when comparing full-term metagenomes from oropharyngeal swabs with our preterm ones.

Despite different initial sampling locations, preterm and full-term specimens were collected, processed and sequenced in the same facility by the same personnel, with identical protocols, kits and negative controls through the same wet-lab and bioinformatics pipelines. Therefore, any center-, protocol-, chemical- or workflow biases should have been avoided including day-to-day variance of contamination [51].

DNA extraction from the low biomass oropharyngeal swabs taken from preterm neonates required quality-controlled working in an ultra-clean environment and both the parallel processing and sequencing of negative controls [18, 52]. This experimental set-up allowed monitoring of the longitudinal dynamics of VLBWs’ microbial airway development by shotgun metagenome sequencing. Heat trees developed by Foster et al. [24] were found to be a valuable tool to visualize the taxonomic composition and allowed a direct comparison of the microbial composition one week postpartum for both elective CS and VD, one month postpartum and after hospital discharge at nine and 15 months of age.

In conclusion, shotgun metagenome sequencing of oropharyngeal swabs from VLBW neonates provided insights into the development of the airway metagenome from one week postpartum to 15 months of age. A profound effect of hospital environment and therapeutic regimens on the metagenome one month postpartum became visible. Fortunately, this effect on alpha and beta diversity vanished at late infancy. However, the low-abundance taxa signature and network structure of bacterial commensals remained fragile in preterm infants. Longer observation periods will be necessary to detect if and when the vulnerable airway community structure of premature born infants will turn to the uniform communities characteristic for healthy children.

## Supplementary information

Supplementary Materials

## Data Availability

The microbial sequencing data are stored in the European Nucleotide Archive (PRJEB52640). Coding R scripts, abundance tables, metadata and a STORMS (Strengthening the Organizing and Reporting of Microbiome Studies) Checklist are available from GitHub (https://github.com/irosenboom/airway_metagenome_preterms).
