# Supplementary material for: Longitudinal development of the airway metagenome of preterm very low birth weight infants during the first two years of life
Source: ISME Commun. 2023 Jul 20;3:75. doi: 10.1038/s43705-023-00285-x (PMC10359316; doi:10.1038/s43705-023-00285-x)
Supplement: Supplementary file 1 — Supplementary Materials [file 43705_2023_285_MOESM1_ESM.pdf]

Longitudinal development of the airway metagenome of preterm very low birth weight infants during the first two years of life

Rosenboom, Ilona[1], Pust, Marie-Madlen[1], Pirr, Sabine[1], Bakker, Alina[1], Willers, Maike[1], Davenport, Colin F.[2], Wiehlmann, Lutz[2], Viemann, Dorothee[1,3,4,5], Tümmler, Burkhard[1]#

[1]Department for Pediatric Pneumology, Allergology and Neonatology, Hannover Medical School, Hannover, Germany

[2]Research Core Unit Genomics, Hannover Medical School, Hannover, Germany

[3]Translational Pediatrics, Department of Pediatrics, University Hospital Würzburg, Würzburg, Germany

[4]Cluster of Excellence RESIST (EXC 2155), Hannover Medical School, Hannover, Germany

[5]Center for Infection Research, University Würzburg, Würzburg, Germany

Running Title: Development of the airway metagenome of VLBW preterms

#Address correspondence to Burkhard Tümmler, tuemmler.burkhard@mh-hannover.de

Supplementary Material

Figures

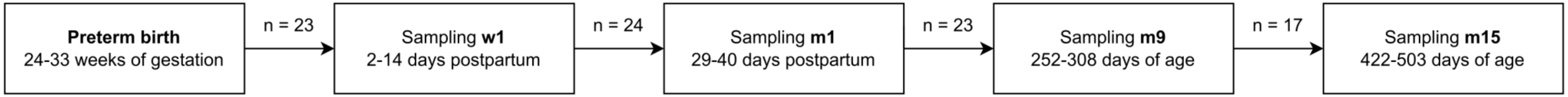

Supplementary Figure S1. Sampling scheme. A scheme indicating the distribution of sampling time points and number of oropharyngeal swabs taken.

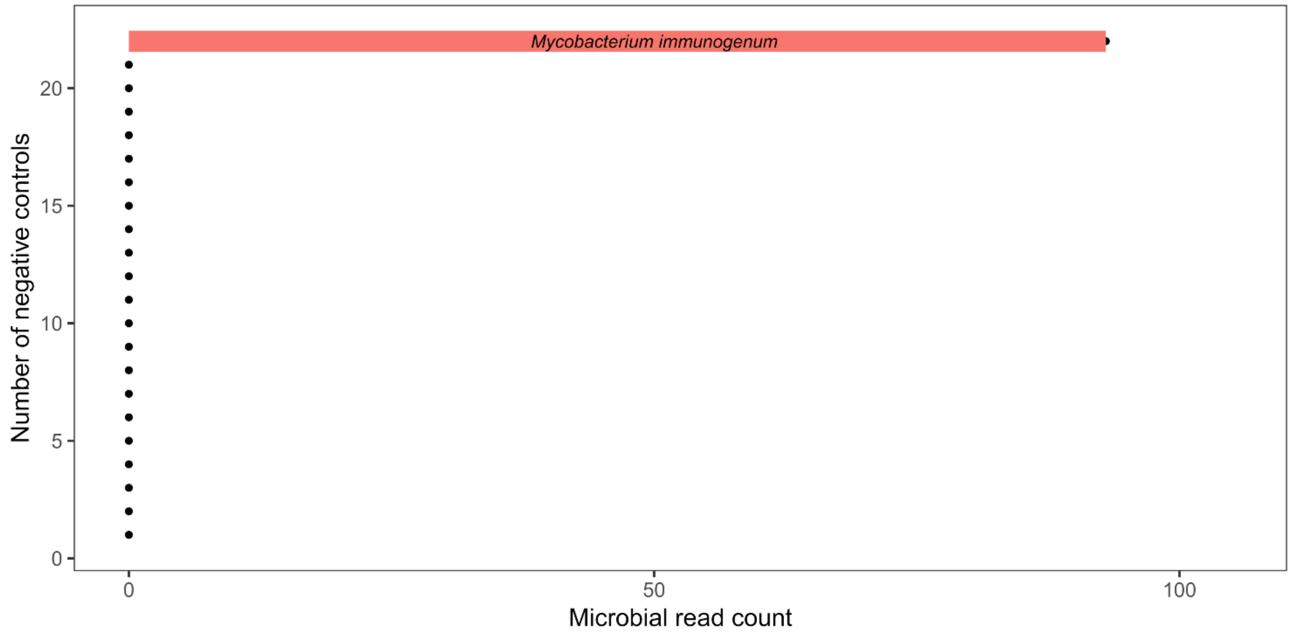

Supplementary Figure S2. Raspir-filtered microbial reads in negative controls. Only one of 22 negative controls revealed bacterial monospecies reads, namely 93 reads of *Mycobacterium immunogenum*. No typical commensals of the respiratory tract were found in the negative controls.

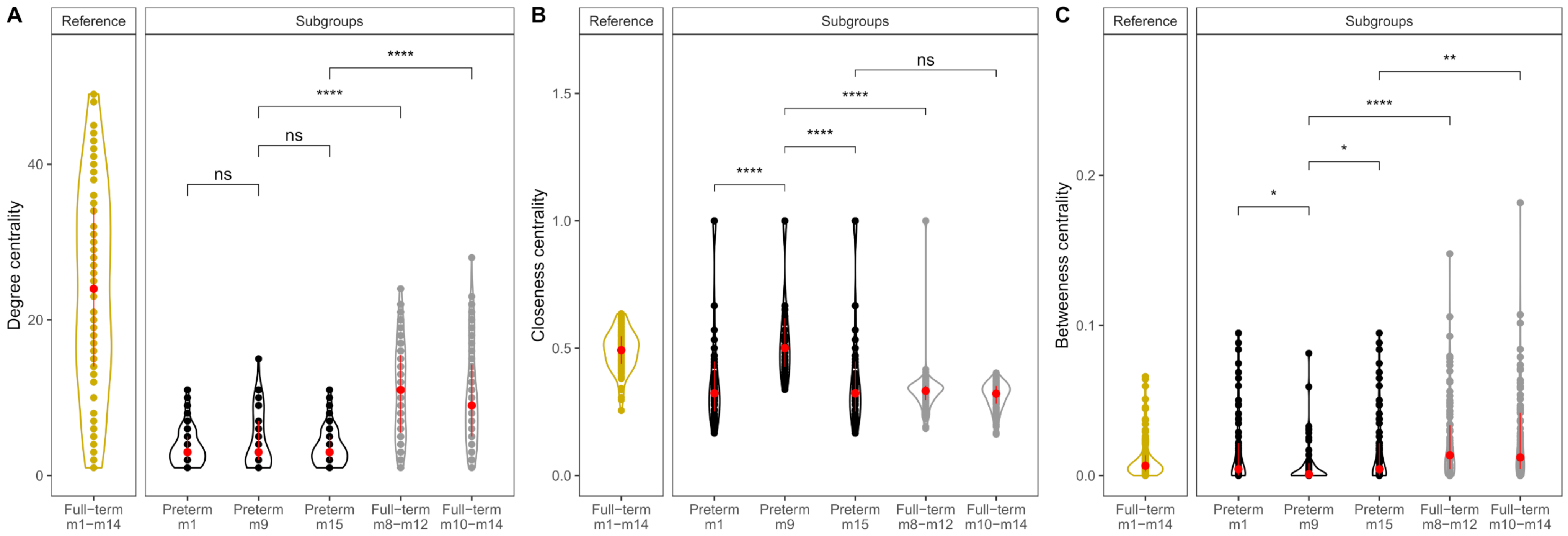

Supplementary Figure S3. Analysis of network parameters within the preterm cohort and comparison with healthy full-term infants. Degree centrality measures the number of connections of a node, Closeness centrality calculates the shortest distance of a node to all other nodes and Betweenness centrality measures how often a node is bridged by the shortest pathway of two other nodes. (A) Comparison of the Degree centrality scores obtained from the species co-occurrence networks per preterm age group and age-matched healthy full-terms (overall Kruskal-Wallis p value < 0.0001, epsilon-squared effect size = 0.45, ci = 0.38-0.53). (B) Comparison of Closeness centrality scores between age-dependent preterm networks and age-matched healthy full-terms (Kruskal-Wallis p value < 0.0001, epsilon-squared effect size = 0.39, ci = 0.33-0.46). (C) Comparison of network Betweenness centrality scores per preterm age group and age-matched healthy full-terms (Kruskal-Wallis p value < 0.0001, epsilon-squared effect size = 0.08, ci = 0.04-0.14). Note: The red dot represents the median, while the red extending lines depict the first (25th percentile) and third quartile (75th percentile). **** p < 0.0001; *** p < 0.001; ** p < 0.01; * p < 0.05; ns, not significant (p > 0.05).

Tables

Table S1. Clinical metadata of study participants and characteristics of longitudinally taken samples.

Patient	Sample	Gender[1]	Gestational age (GA)	Birth weight (grams)	Mode of delivery[2]	Age (in days post-partum)	Age (in days after gestation)	Age corrected for full-term pregnancy (in days)[3]	Feeding[4]
19_003a	w1	f	33	1910	elec. CS	2	233	-47	BM + F
19_003b	m1	f	33	1910	elec. CS	30	261	-19	BM + F
19_003c	m9	f	33	1910	elec. CS	252	483	203	F + CK
19_003d	m15	f	33	1910	elec. CS	422	653	373	F + CK + FF
19_004a	w1	f	33	1770	elec. CS	2	233	-47	BM + F
19_004b	m1	f	33	1770	elec. CS	30	261	-19	BM + F
19_004c	m9	f	33	1770	elec. CS	252	483	203	F + CK
19_004d	m15	f	33	1770	elec. CS	422	653	373	F + CK + FF
19_013b	m1	m	26	640	VD	29	211	-69	BM + F
19_013c	m9	m	26	640	VD	289	471	191	F + CK
19_013d	m15	m	26	640	VD	477	659	379	F + CK
19_020a	w1	f	29	1220	VD	4	207	-73	BM + F
19_020b	m1	f	29	1220	VD	32	235	-45	BM + F
19_020c	m9	f	29	1220	VD	295	498	218	F + CK
19_027a	w1	m	30	1015	non-elec. CS	4	214	-66	BM + F
19_027b	m1	m	30	1015	non-elec. CS	36	246	-34	BM + F
19_027c	m9	m	30	1015	non-elec. CS	267	477	197	F
19_027d	m15	m	30	1015	non-elec. CS	450	660	380	FF
19_028a	w1	f	28	860	non-elec. CS	8	204	-76	BM + F
19_028b	m1	f	28	860	non-elec. CS	33	229	-51	BM + F
19_028c	m9	f	28	860	non-elec. CS	272	468	188	BM + F
19_028d	m15	f	28	860	non-elec. CS	503	699	419	BM + F
19_029a	w1	m	28	930	non-elec. CS	8	204	-76	BM + F
19_029b	m1	m	28	930	non-elec. CS	33	229	-51	BM + F
19_029c	m9	m	28	930	non-elec. CS	272	468	188	BM + F
19_029d	m15	m	28	930	non-elec. CS	503	699	419	BM + F
19_030a	w1	m	28	585	non-elec. CS	8	204	-76	BM + F
19_030b	m1	m	28	585	non-elec. CS	33	229	-51	BM + F
19_030c	m9	m	28	585	non-elec. CS	272	468	188	BM + F
19_030d	m15	m	28	585	non-elec. CS	503	699	419	BM + F
19_033a	w1	m	31	1375	VD	2	219	-61	BM + F
19_033b	m1	m	31	1375	VD	29	246	-34	BM + F

19_033c	m9	m	31	1375	VD	261	478	198	F + CK
19_033d	m15	m	31	1375	VD	442	659	379	F + CK + FF
19_037a	w1	f	30	1400	non-elec. CS	3	213	-67	BM + F
19_037b	m1	f	30	1400	non-elec. CS	33	243	-37	BM + F
19_037c	m9	f	30	1400	non-elec. CS	255	465	185	BM + F
19_037d	m15	f	30	1400	non-elec. CS	452	662	382	BM + F + CK + FF
19_041a	w1	f	27	795	non-elec. CS	5	194	-86	BM + F
19_041b	m1	f	27	795	non-elec. CS	30	219	-61	BM + F
19_041c	m9	f	27	795	non-elec. CS	289	478	198	BM + CF + FF
19_041d	m15	f	27	795	non-elec. CS	465	654	374	FF
19_046a	w1	m	29	995	elec. CS	2	205	-75	BM + F
19_046b	m1	m	29	995	elec. CS	34	237	-43	BM + F
19_046c	m9	m	29	995	elec. CS	278	481	201	FF
19_046d	m15	m	29	995	elec. CS	448	651	371	FF
19_050a	w1	f	31	640	elec. CS	4	221	-59	BM + F
19_050b	m1	f	31	640	elec. CS	40	257	-23	BM + F
19_050c	m9	f	31	640	elec. CS	260	477	197	BM + CF
19_050d	m15	f	31	640	elec. CS	436	653	373	FF
19_053a	w1	f	29	720	elec. CS	2	205	-75	BM + F
19_053b	m1	f	29	720	elec. CS	36	239	-41	BM + F
20_004a	w1	f	28	655	elec. CS	4	200	-80	BM + F
20_004b	m1	f	28	655	elec. CS	36	232	-48	BM + F
20_004c	m9	f	28	655	elec. CS	289	485	205	F + CK + FF
20_004d	m15	f	28	655	elec. CS	463	659	379	F + FF
20_017a	w1	m	26	800	VD	4	186	-94	F
20_017b	m1	m	26	800	VD	40	222	-58	F
20_017c	m9	m	26	800	VD	305	487	207	F + CK
20_017d	m15	m	26	800	VD	471	653	373	F + CK + FF
20_021a	w1	f	29	990	non-elec. CS	5	208	-72	BM + F
20_021b	m1	f	29	990	non-elec. CS	29	232	-48	BM + F
20_021c	m9	f	29	990	non-elec. CS	266	469	189	BM + F
20_021d	m15	f	29	990	non-elec. CS	448	651	371	F + CK
20_022a	w1	m	29	1220	non-elec. CS	6	209	-71	BM + F
20_022b	m1	m	29	1220	non-elec. CS	29	232	-48	BM + F
20_022c	m9	m	29	1220	non-elec. CS	266	469	189	BM + F
20_022d	m15	m	29	1220	non-elec. CS	448	651	371	F + CK
20_023a	w1	f	29	1235	non-elec. CS	6	209	-71	BM + F
20_023b	m1	f	29	1235	non-elec. CS	29	232	-48	BM + F
20_023c	m9	f	29	1235	non-elec. CS	266	469	189	BM + F
20_023d	m15	f	29	1235	non-elec. CS	448	651	371	F + CK

20_028a	w1	m	24	655	VD	14	182	-98	BM + F
20_028b	m1	m	24	655	VD	35	203	-77	BM + F
20_028c	m9	m	24	655	VD	308	476	196	F + CK
20_041a	w1	f	27	975	non-elec. CS	4	193	-87	BM + F
20_041b	m1	f	27	975	non-elec. CS	35	224	-56	BM + F
20_041c	m9	f	27	975	non-elec. CS	282	471	191	F + CK
20_052a	w1	f	26	890	VD	6	188	-92	BM + F
20_052b	m1	f	26	890	VD	31	213	-67	BM + F
20_052c	m9	f	26	890	VD	302	484	204	F + CK
20_058a	w1	f	29	1080	VD	4	207	-73	BM + F
20_058b	m1	f	29	1080	VD	29	232	-48	BM + F
20_058c	m9	f	29	1080	VD	264	467	187	F + CK
20_061a	w1	m	27	940	non-elec. CS	3	192	-88	BM + F
20_061b	m1	m	27	940	non-elec. CS	37	226	-54	BM + F
20_061c	m9	m	27	940	non-elec. CS	276	465	185	F + CK

[1] Gender: f (female), m (male)
[2] Mode of delivery: elec. CS (elective Caesarean section), non-elec. CS (non-elective Caesarean section), VD (vaginal delivery)
[3] Age corrected for full-term pregnancy: Age (in days after gestation) – 280 days (full pregnancy)
[4] Feeding: BM (breast milk), F (formula), CF (complementary feeding), FF (family food)

Table S2. Clinical metadata and information on treatment of study participants.

Patient	Sample	Group	Ventilation	Antimicrobial therapy in neonatal period	Medication (treatment length in days)*	Episodes of antimicrobial therapy after hospital discharge during the first year of life
19_003a	w1	non-BPD	CPAP	untreated	Caffeine (9d), Xylometazoline (3d)	once
19_003b	m1	non-BPD	none			
19_003c	m9	non-BPD	none			
19_003d	m15	non-BPD	none			
19_004a	w1	non-BPD	CPAP	untreated	Caffeine (19d)	once
19_004b	m1	non-BPD	none			
19_004c	m9	non-BPD	none			
19_004d	m15	non-BPD	none			
19_013b	m1	BPD	intubation	treated	Surfactant, Caffeine (78d), Doxapram (5d), Furosemide (4d), L-Thyroxin (40d), Nystatin (3d), Fluconazole (5d), Xylometazoline (6d)	once
19_013c	m9	BPD	none			
19_013d	m15	BPD	none			
19_020a	w1	non-BPD	CPAP	untreated	Caffeine (41d), Xylometazoline	untreated
19_020b	m1	non-BPD	none			
19_020c	m9	non-BPD	none			
19_027a	w1	non-BPD	CPAP	treated	Surfactant, Caffeine (33d), L-Thyroxin (27d)	untreated
19_027b	m1	non-BPD	none			
19_027c	m9	non-BPD	none			
19_027d	m15	non-BPD	none			
19_028a	w1	non-BPD	CPAP	treated	Surfactant, Caffeine (45d)	untreated
19_028b	m1	non-BPD	CPAP			
19_028c	m9	non-BPD	none			
19_028d	m15	non-BPD	none			
19_029a	w1	non-BPD	CPAP	treated	Caffeine (45d), Indomethacin (4d)	untreated
19_029b	m1	non-BPD	CPAP			
19_029c	m9	non-BPD	none			
19_029d	m15	non-BPD	none			
19_030a	w1	BPD	CPAP	treated	Surfactant, Caffeine (4d), Indomethacin	untreated
19_030b	m1	BPD	CPAP			
19_030c	m9	BPD	none			
19_030d	m15	BPD	none			
19_033a	w1	non-BPD	CPAP	untreated	Caffeine	
19_033b	m1	non-BPD	none			

19_033c	m9	non-BPD	none			untreated
19_033d	m15	non-BPD	none			
19_037a	w1	non-BPD	intubation	treated	Surfactant, Caffeine (29d), Catecholamines (3d)	
19_037b	m1	non-BPD	none			
19_037c	m9	non-BPD	none			once
19_037d	m15	non-BPD	none			
19_041a	w1	BPD	CPAP	treated	Surfactant, Caffeine (52d), Fluconazole (7d)	
19_041b	m1	BPD	CPAP			
19_041c	m9	BPD	none			untreated
19_041d	m15	BPD	none			
19_046a	w1	BPD	intubation	treated	Surfactant, Caffeine (56d)	
19_046b	m1	BPD	high-flow			
19_046c	m9	BPD	none			once
19_046d	m15	BPD	none			
19_050a	w1	BPD	CPAP	treated	Caffeine (26d)	
19_050b	m1	BPD	low-flow			
19_050c	m9	BPD	none			untreated
19_050d	m15	BPD	none			
19_053a	w1	BPD	intubation	treated	Surfactant, Caffeine	-
19_053b	m1	BPD	CPAP			
20_004a	w1	BPD	intubation	treated	Surfactant, Caffeine (63d), Catecholamines (4d)	
20_004b	m1	BPD	CPAP			
20_004c	m9	BPD	none			untreated
20_004d	m15	BPD	none			
20_017a	w1	BPD	intubation	treated	Surfactant, Caffeine, Dexamethasone, Methylprednisolone, Indomethacin (5d)	
20_017b	m1	BPD	CPAP			
20_017c	m9	BPD	none			untreated
20_017d	m15	BPD	none			
20_021a	w1	non-BPD	CPAP	untreated	Caffeine (39d)	
20_021b	m1	non-BPD	low-flow			
20_021c	m9	non-BPD	none			untreated
20_021d	m15	non-BPD	none			
20_022a	w1	BPD	CPAP	untreated	Caffeine (44d), Doxapram (1d)	
20_022b	m1	BPD	low-flow			
20_022c	m9	BPD	none			untreated
20_022d	m15	BPD	none			
20_023a	w1	non-BPD	CPAP	untreated	Caffeine (36d), Indomethacin (5d)	
20_023b	m1	non-BPD	low-flow			
20_023c	m9	non-BPD	none			untreated
20_023d	m15	non-BPD	none			

20_028a	w1	BPD	CPAP	treated	Surfactant, Caffeine (86d), Catecholamines (2d) , Doxapram (2d), Prednisolone (22d), Dexamethasone (22d), Indomethacin (8d)	
20_028b	m1	BPD	CPAP			
20_028c	m9	BPD	none			untreated
20_041a	w1	BPD	CPAP	treated	Surfactant, Caffeine	
20_041b	m1	BPD	CPAP			
20_041c	m9	BPD	none			untreated
20_052a	w1	BPD	CPAP	treated	Surfactant, Caffeine (58d), Nystatin (5d)	
20_052b	m1	BPD	CPAP			
20_052c	m9	BPD	none			once
20_058a	w1	non-BPD	CPAP	treated	Caffeine (54d), Mometasone (8d)	
20_058b	m1	non-BPD	CPAP			
20_058c	m9	non-BPD	none			untreated
20_061a	w1	non-BPD	CPAP	treated	Surfactant, Caffeine (53d), Doxapram (15d), Indomethacin (5d), Heparine (1x)	
20_061b	m1	non-BPD	CPAP			
20_061c	m9	non-BPD	none			untreated

*Data on treatment length is missing if information could not be retrieved from the patient’s charts.

Table S3. Clinical metadata of healthy full-term infants.

Infant ID	Gender	Age (in days post-partum)	Antimicrobial therapy
KGCF32	m	37	untreated
KGCF07	m	63	untreated
KGCF10	m	67	untreated
KGCF47	f	97	untreated
KGCF18	m	104	untreated
KGCF43	f	132	untreated
KGCF14	m	139	untreated
KGCF46	m	163	untreated
KGCF02	m	165	untreated
KGCF42	m	174	untreated
KGCF52	m	189	untreated
KGCF12	m	193	untreated
KGCF39	m	198	untreated
KGCF17	m	213	untreated
KGCF56	f	239	untreated
KGCF41	f	243	untreated
KGCF49	m	248	untreated
KGCF48	f	251	untreated
KGCF44	f	255	untreated
KGCF16	f	269	untreated
KGCF53	m	285	untreated
KGCF58	f	289	untreated
KGCF57	f	291	untreated
KGCF51	m	307	untreated
KGCF45	m	330	untreated
KGCF55	f	344	untreated
KGCF04	m	353	untreated
KGCF36	f	364	untreated
KGCF11	m	398	untreated
KGCF50	m	398	untreated
*Samples were taken from a previously published study (18).			

Supplementary Table S4. Permutation tests of non-metric multidimensional scaling based on Bray-Curtis dissimilarity indices. Clinical variables and species were fitted in the ordination (*envfit*).

A. Preterm cohort.

Variables	Goodness of fit (r^2)	Goodness of fit (p)
Group (BPD vs non-BPD)	0.016	0.286
Age (in days)	0.675	0.001 ***
Species number	0.693	0.001 ***
Shannon diversity	0.712	0.001 ***
Ventilation mode	0.648	0.001 ***
Antimicrobial therapy in neonatal period	0.016	0.522

B. Preterm m15 and healthy full-term infants.

Variables	Goodness of fit (r^2)	Goodness of fit (p)
Group (preterm vs healthy infant)	0.214	0.003 **
Age (in days)	0.325	0.008 **
Species number	0.897	0.001 ***
Shannon diversity	0.198	0.065
Antimicrobial therapy	0.053	0.244

Supplementary Table S5. Permutational Multivariate Analysis of Variance (PERMANOVA) using Bray-Curtis distance matrices. Displayed are results on clinical metadata in both the preterm cohort only and a comparison of m15 preterm infants with age-matched healthy full-terms (Df = degrees of freedom, SumofSqs = sum of squares, F Model = F value by permutation, R^2 and *p* values are based on 999 permutations).

A. Preterm cohort.

Factors	Df	SumofSqs	F Model	R^2	*p* value
Age					
Age	3	6.004	5.376	0.170	0.001 ***
Residuals	79	29.411		0.830	
Total	82	35.415		1	
Ventilation mode					
Ventilation mode	1	3.342	8.4414	0.094	0.001 ***
Residuals	81	32.073		0.906	
Total	82	35.415		1	
Group (BPD – non-BPD)					
Group	1	0.449	1.0403	0.013	0.353
Residuals	81	34.966		0.987	
Total	82	35.415		1	
Antimicrobial therapy in neonatal period					
Therapy	1	0.642	1.4945	0.018	0.063
Residuals	81	34.773		0.982	
Total	82	35.415		1	
Species number					
Species Number	1	4.485	11.745	0.127	0.001 ***
Residuals	81	30.930		0.873	
Total	82	34.451		1	
Shannon diversity					
Shannon diversity	1	4.191	10.873	0.118	0.001 ***
Residuals	81	31.224		0.882	
Total	82	35.415		1	

B. Preterm m15 and healthy full-term infants.

Factors	Df	SumsofSqs	F Model	R^2	*p* value
Age					
Age	1	0.4350	1.4864	0.056	0.125
Residuals	25	7.3165			
Total	26	7.7515			
Group (Preterm – healthy full-term)					
Group	1	0.7503	2.6792	0.097	0.004 **
Residuals	25	7.0		0.903	
Total	26	7.7515		1	
Antimicrobial therapy					
Therapy	1	0.3989	1.3562	0.051	0.173
Residuals	25	7.3526		0.949	
Total	26	7.7515		1	
Species number					
Species Number	1	1.2734	4.9145	0.164	0.001 ***
Residuals	25	6.4780		0.836	
Total	26	7.7515		1	
Shannon diversity					
Shannon diversity	1	0.8445	3.0567	0.109	0.004 **
Residuals	25	6.9070		0.891	
Total	26	7.7515		1	
